# Advancing Orthodontic Aesthetics: Exploring the Potential of Injectable Composite Resin Techniques for Enhanced Smile Transformations

**DOI:** 10.3390/dj13010018

**Published:** 2024-12-30

**Authors:** Davide Spadoni, Cristina Valeri, Vincenzo Quinzi, Ute Schneider Moser, Giuseppe Marzo

**Affiliations:** 1 Independent Researcher, 19020 La Spezia, Italy; dottspadoni.davide@libero.it; 2 Department of Life, Health and Environmental Sciences, Postgraduate School of Orthodontics, University of L’Aquila, 67100 L’Aquila, Italy; vincenzo.quinzi@univaq.it (V.Q.); giuseppe.marzo@univaq.it (G.M.); 3 Department of Orthodontics, University of Ferrara, 44121 Ferrara, Italy; ute.schneider-moser@outlook.de; 4 Department of Orthodontics, School of Dental Medicine, University of Pennsylvania, Philadelphia, PA 19104, USA

**Keywords:** injection moulding technique, high-filled composite, injectable composite, clear silicon index, digital flow, diagnostic wax-up

## Abstract

**Background/Objectives:** The injection moulding technique (IMT) is a minimally invasive restorative treatment. This technique enables the application of thin, flowable composite layers into a stable, transparent silicone index that serves as a mould. Due to the fluid properties of the composite, it efficiently fills the silicone tray and seamlessly integrates with the tooth structure, often obviating tooth preparation and preserving overall tooth integrity. The procedure employs the etch-and-rinse protocol and is highly reproducible. Minimally invasive restorative techniques are particularly relevant following orthodontic treatment, where minor tooth adjustments are often required to achieve optimal aesthetics and function. Integrating orthodontic and restorative treatments is pivotal for long-term success, especially in complex interdisciplinary cases. **Methods:** This retrospective study describes the application of conservative restoration using the IMT in two pediatric patients (12.6 years old and 12.3 years old) to restore maxillary lateral incisors before and after orthodontic treatment. The technique provides a viable option for temporary composite restorations until the patients are suitable candidates for permanent all-ceramic veneers. **Results:** The injectable technique is ideal for minimal diastemas, small interdental spaces, or retruded teeth. The cases presented, involving irregular tooth sizes and morphologies, demonstrate the suitability of the IMT in scenarios requiring an additive approach. This technique effectively addresses such irregularities without necessitating invasive preparation. **Conclusions:** The IMT is a valuable tool for pediatric patients undergoing orthodontic treatment, both at its initiation and completion. The technique assists orthodontists in finalising treatment by addressing Bolton index discrepancies and correcting tooth shape anomalies. Additionally, a digital workflow reduces clinical sessions, as thermo-printed retainers can be delivered during the same appointment as the IMT, providing economic and organisational benefits. This approach underscores the utility of the IMT in enhancing treatment efficiency and outcomes in orthodontic–restorative care.

## 1. Introduction

In clinical dental practice, aesthetics is increasingly recognised as a critical aspect of patient care [1]. Modern dental rehabilitation addresses occlusal functionality and aims to achieve comprehensive restoration, enhancing the individual’s overall well-being, quality of life, and self-confidence [2]. Various approaches exist for rehabilitating a patient’s smile, which depends on factors such as the patient’s age and specific clinical considerations. Among these, ceramic veneers are highly favoured due to their proven efficacy, superior aesthetic results, and favourable biomechanical properties in various clinical scenarios [3,4,5]. Composite restorations have also become widely utilised in operative, prosthetic, and aesthetic dentistry [6], offering significant advantages in reduced time and cost compared to traditional prosthetic solutions [7,8,9,10,11].

Using the freehand bonding method in aesthetic restorative procedures involves precisely layering composite materials with varying opacities. This technique achieves excellent aesthetic outcomes and long-term clinical success, providing a cost-effective and conservative therapeutic alternative [12]. However, it requires high expertise, advanced clinical skills, and a significant time investment during patient-chair sessions [13].

Implementing computer-aided design and manufacturing (CAD/CAM) techniques in dentistry has equipped clinicians with innovative tools for managing diverse clinical situations [14]. Contemporary digital technologies such as intraoral scanning, cone-beam computed tomography (CBCT), digital smile design (DSD), and digital orthodontic setups facilitate 3D planning for interdisciplinary treatments [15]. Digital pre-visualization, especially DSD, is pivotal in early treatment planning, guiding the final restorative phases by defining desired tooth aesthetics and function [16]. Open-source software (Digital Smile Design—Keynote) further enhances digital design efficiency and cost-effectiveness during pre-visualisation [17,18].

The injection moulding technique (IMT) offers a practical alternative to overcome the limitations of freehand bonding. The IMT involves introducing a flowable composite material into a stable, transparent silicone index that serves as a mould [7,8,9,10]. The composite material’s fluid properties allow it to fill the silicone index effectively and seamlessly integrate it with the tooth structure. These rheological characteristics enable the application of thin layers, often eliminating the need for tooth preparation and preserving overall tooth integrity. The etch-and-rinse protocol, a cornerstone of this technique, ensures consistency and repeatability [10].

After orthodontic treatment, orthodontists can utilise minimally invasive restorations to perform minor modifications to tooth form, thereby achieving optimal aesthetics and functionality. Integrating orthodontic and restorative treatments is crucial for maintaining favourable therapeutic outcomes, particularly in interdisciplinary cases. Conservative interventions are especially beneficial in addressing conditions such as tooth wear, incisal margin abrasions, tooth shape or size irregularities, and significant Bolton discrepancies [7].

This study outlines the use of the IMT for conservative tooth restoration in pediatric patients undergoing orthodontic treatment, specifically for restoring maxillary lateral incisors. The IMT is a viable temporary solution until the patient qualifies for permanent all-ceramic veneers. Using a digital workflow, orthodontists can efficiently design the desired tooth morphology. Thermoformed retainers or aligners can then be fabricated during the same mock-up phase and delivered to the patient following the completion of tooth reshaping, optimising the efficiency of patient-chair sessions. The specific clinical requirements of the cases guided the decision to utilize the IMT over 3D-printing methods. While 3D-printing technologies offer precise and customisable solutions, the IMT provides a more time-efficient approach with comparable aesthetic and functional results. Furthermore, the simplicity and cost-effectiveness of the IMT make it particularly advantageous for pediatric cases, where minimally invasive and conservative interventions are paramount. The straightforward integration of the IMT with traditional orthodontic workflows also enhances its accessibility and practicality in routine clinical settings.

## 2. Case Presentation

These case reports adhere to the 2013 CARE checklist [19]. All subjects provided informed consent before they participated in the study. The study was conducted in accordance with the Declaration of Helsinki, and the protocol received approval from the University of L’Aquila Ethics Committee (L’Aquila, Italy) (Project identification code: 12/2020; date of approval: 11 May 2020).

### 2.1. Case 1

#### 2.1.1. Patient Presentation

A 12.6-year-old female patient presented for an orthodontic consultation due to dissatisfaction with the aesthetic appearance of her smile. Clinical examination revealed that her upper lateral incisors (teeth 12 and 22) exhibited reduced dimensions and atypical morphology.

A detailed patient history was obtained, which indicated no underlying medical conditions and adherence to a healthy lifestyle. A caries risk assessment was performed, including an evaluation of the patient’s dietary habits, which were found to reflect a balanced diet.

#### 2.1.2. Clinical Findings

The patient presented with a skeletal Class I relationship and an oval facial configuration characterised by symmetrical proportions and a convex profile. The molars and canines exhibited a Class I relationship, and the dental midlines aligned well. No carious lesions were observed in either the anterior or posterior dentition. Mild misalignment was noted in the lower dental arch.

#### 2.1.3. Diagnosis and Assessment

**Figure 1:**
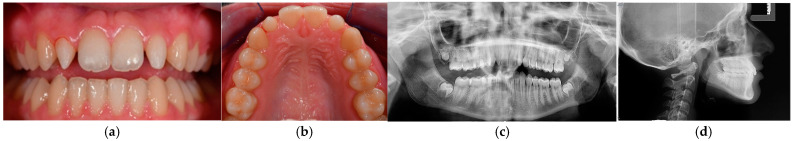
(**a**) Frontal view of teeth with a slightly open mouth to elucidate the conoidal teeth (12 and 22). (**b**) Occlusal perspective of the patient’s maxillary arch. (**c**,**d**) Radiographic evaluations.

The diagnostic process involved collecting conventional orthodontic records, including photographic documentation, radiographic imaging, and dental casts. These records were systematically organised and analysed using digital software (Digital Smile Design—Keynote) platforms to ensure comprehensive data management (Figure 1). Subsequently, the software was utilised to assess and modify tooth dimensions and morphology digitally.

#### 2.1.4. Intervention Types Considered

The orthodontist evaluated two treatment strategies as potential solutions for the patient’s malocclusion and compromised smile aesthetics. The first strategy involved extracting the crowded lower incisor (tooth 42) to address the crowding issue and closing the spaces in the upper arch through direct or indirect reconstructions of teeth 22 and 12. This approach required a fixed upper retainer and a lower fixed retainer spanning from tooth 33 to tooth 43. The second option avoided tooth extraction and proposed creating space within the lower arch through interproximal enamel reduction.

This second approach involved utilising the IMT to reconstruct teeth 12 and 22, followed by removable upper retainers and fixed lower retainers extending from tooth 43 to tooth 33. The patient’s preferences guided the selection of the second option as the treatment of choice.

#### 2.1.5. Therapeutic Intervention

**Figure 2:**
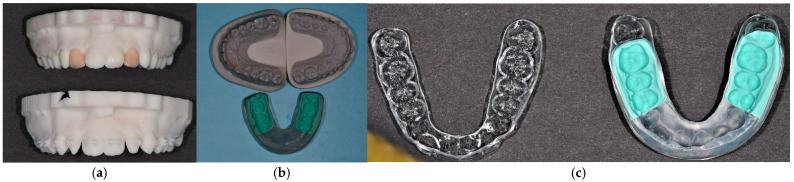
(**a**) Wax-up of teeth 12 and 22. (**b**) Thermo-printed retainer placed on the upper left model. The top right exhibits the final model following orthodontic treatment, while the model employed for generating the triple-layer silicone index is depicted on the bottom. (**c**) Thermo-printed retainer on the left and triple-layer silicone index on the right, both derived from the same digital planning process.

Following finalising the desired tooth morphology using digital software, a preliminary assessment involved placing a provisional resin mock-up in the patient’s oral cavity. This mock-up was tailored to meet the patient’s aesthetic and functional needs. The creation of the definitive mould was contingent upon achieving satisfactory results for the patient and the clinician. Subsequently, a thermally moulded plastic tray enveloping a silicone stent was fabricated in alignment with the final wax-up. The thermo-printed retainer and triple-layer silicone were also derived from the same digital planning process (Figure 2).

**Figure 3:**
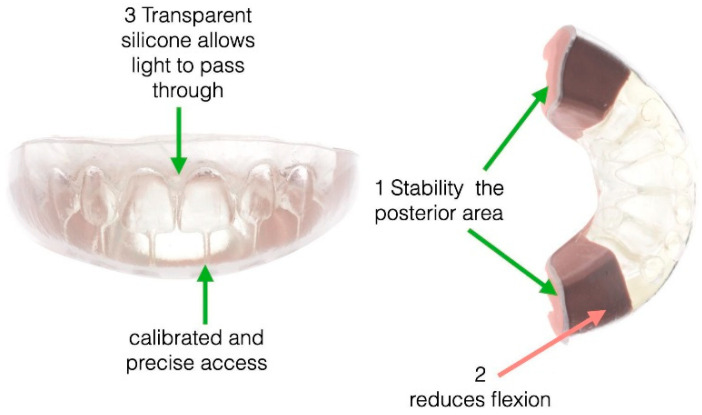
The three-layer silicon index’s parts.

A triple-layer transparent silicone index was employed to precisely replicate the diagnostic wax-up for direct composite restorations. This index consisted of three specific layers: Index 1 provided stability in the posterior region, Index 2 minimised flexural effects, and Index 3 facilitated light transmission. Strategically placed holes at the incisal edges allowed for a calibrated and precise pathway for the composite syringe (Figure 3). The transparent silicone material provided an optimal balance of flexibility and stability during moulding. The triple-layer index was fabricated using EXACLEAR GC Transparent Silicone for moulding templates (durometer hardness ~60 Shore-A), ERCO-DURE thermoformed plates (2–3 mm thickness) for final retention and mask stabilisation, and LASCODE ERGASIL Double Paste A + B Silicone (70 Shore-A hardness).

**Figure 4:**
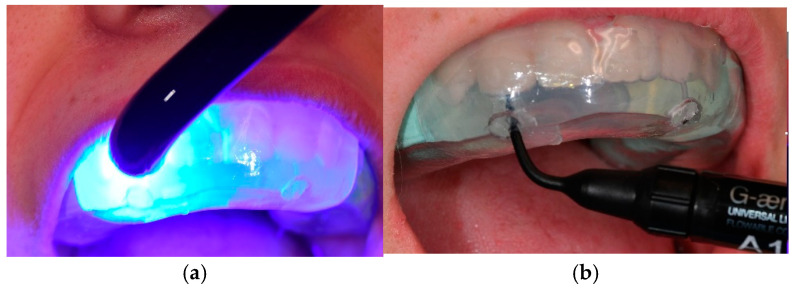
(**a**) Gradual application of flowable composite material. (**b**) Light-curing procedure.

The teeth were etched and treated with a bonding agent. A gradual application of A1 shade flowable composite material (G-ænial Universal Injectable, GC America, Illinois, USA) was conducted using the silicone index to achieve the desired tooth contours. Following light curing (Elipar ™ Deepcure-L LED Curing Light, 3M - Minnesota, USA; wavelength: 430–480 nm; light intensity: 1470 mW/cm^2^), the excess composite material was meticulously removed using a bur or scalpel to prevent overhangs that could lead to plaque accumulation and periodontal irritation. A comprehensive polishing regimen ensured the longevity of the restorations. Finishing tools included fine-grit flame-shaped burs (Komet Dental—Lemgo, Germany, green), 3M discs of varying grit levels, and finishing tips for the Soniflex handpiece (Kavo—Biberach, Germany). Polishing was completed using a goat hair wheel, diamond polishing paste, and Sof-Lex spirals to enhance the surface shine of the composite resin (Figure 4). 

**Figure 5:**
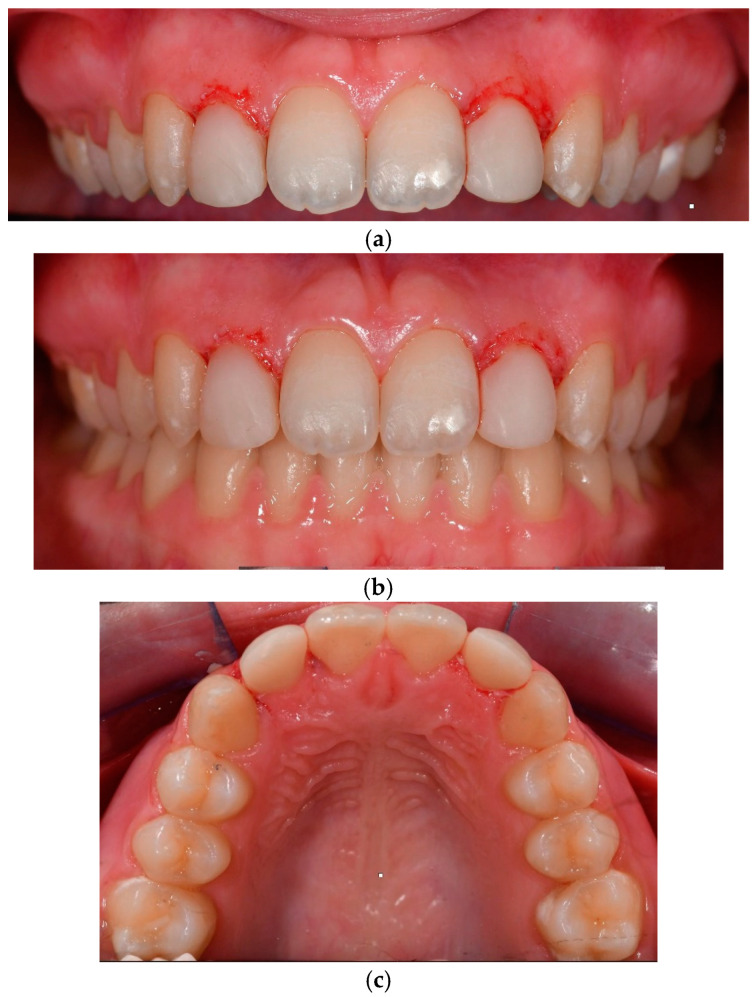
(**a**) Detailed reshaping of teeth 12 and 22. (**b**) Frontal view of final occlusion. (**c**) Occlusal view to highlight the palatal aspect of the reconstructions on 12 and 22.

The therapeutic intervention spanned 22 months and concluded with the patient’s satisfaction regarding her enhanced smile (Figure 5). The digital workflow facilitated the retainer delivery at the end of the conservative procedure.

**Figure 6:**
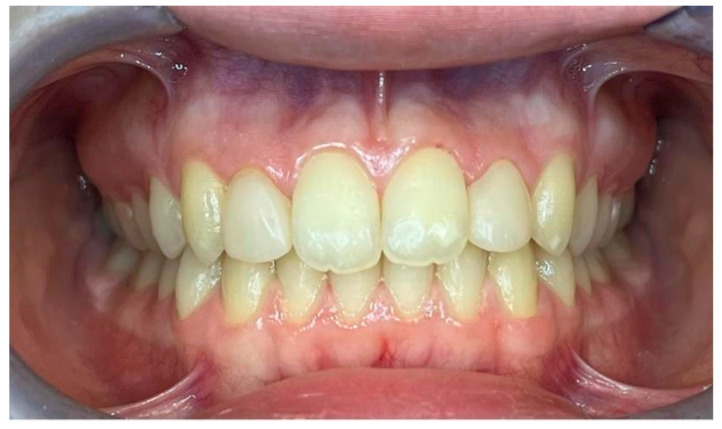
Frontal view of final occlusion in a six-year follow-up control.

A six-month post-treatment evaluation revealed no signs of gingival inflammation, preserved periodontal health, and stable colour retention of teeth 12 and 22. The patient exhibited high tolerance and acceptance of the procedure. A six-year follow-up examination demonstrated no bleeding, physiological probing depths, healthy periodontal conditions, and satisfactory oral hygiene (Figure 6).

### 2.2. Case 2

#### 2.2.1. Patient Presentation

**Figure 7:**
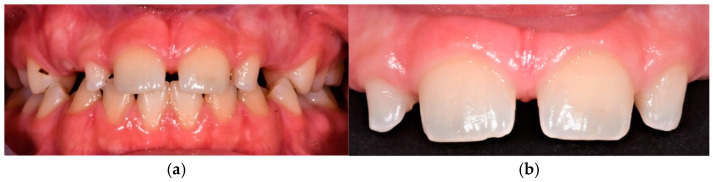
(**a**) Frontal view of the dental occlusion in maximal intercuspation. (**b**) Detailed dental anomalies of teeth 12 and 22.

A 12.3-year-old male patient presented for an orthodontic consultation due to dissatisfaction with the aesthetic appearance of his smile. Clinical examination revealed small lateral incisors that were not prominent and unerupted canines (teeth 13 and 23) (Figure 7).

The patient provided a comprehensive medical history, confirming the absence of concurrent medical conditions and adherence to a healthy lifestyle. The caries risk assessment included an evaluation of the patient’s dietary habits, which were determined to be healthy.

#### 2.2.2. Clinical Findings

The patient exhibited a skeletal Class I relationship with a tendency toward Class III and an oval facial form characterised by symmetrical proportions and a convex profile—the molar and canine relationships aligned in a Class I configuration with harmonious midline alignment (Figure 7). Dental arches displayed spacing without crowding, and no carious lesions were observed in the anterior or posterior regions.

#### 2.2.3. Diagnosis and Assessment

**Figure 8:**
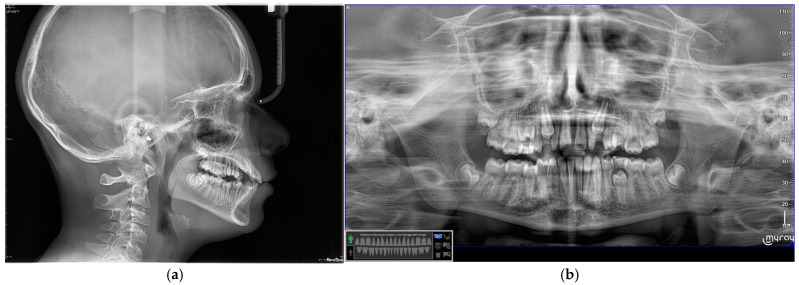
(**a**,**b**) Radiographic examination.

Diagnostic records were obtained and analysed, including photographic documentation, radiographic imaging, and dental models (Figure 8). Proprietary or open-source software applications were employed to modify tooth dimensions and morphology digitally.

#### 2.2.4. Intervention Types Considered

Two therapeutic approaches were evaluated to address the patient’s malocclusion and aesthetic concerns. The first option involved space closure and correction of the Class III tendency. The second approach proposed a direct restoration for teeth 12 and 22 during the pre-orthodontic phase, followed by a 24-month orthodontic treatment plan, space closure, and final upper and lower retainer application. The patient selected the second approach based on his preferences.

#### 2.2.5. Therapeutic Intervention

The desired tooth morphology was finalised using digital methods, and an initial temporary resin mock-up was placed in the patient’s mouth. Adjustments were made to meet the patient’s preferences.

**Figure 9:**
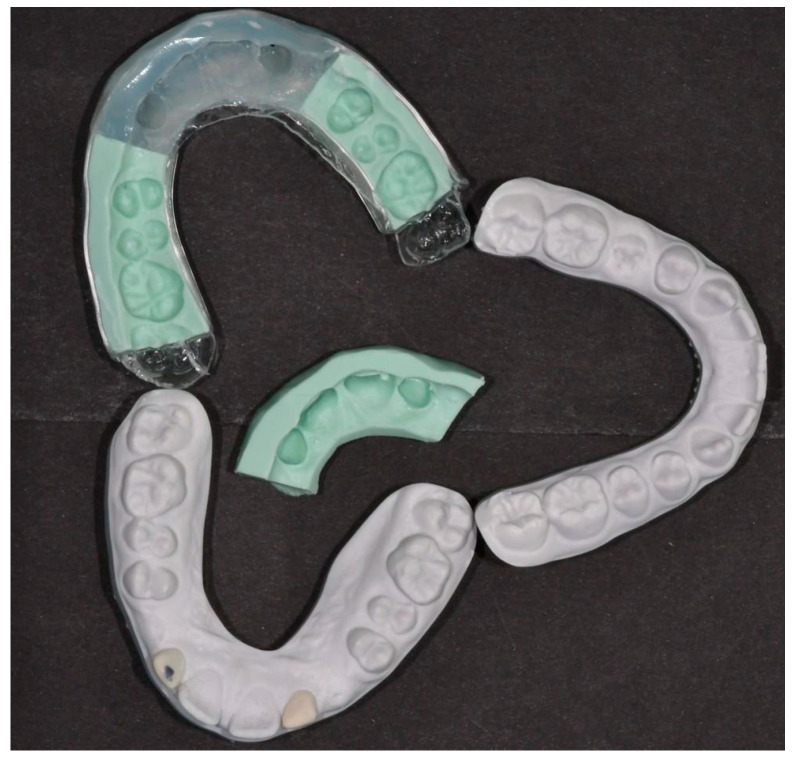
Thermo-printed retainer placed on the silicon tray on the upper left side. The bottom left exhibits the final model with the wax-up employed for generating the triple-layer silicon index. The model on the left represents the lower arch.

**Figure 10:**
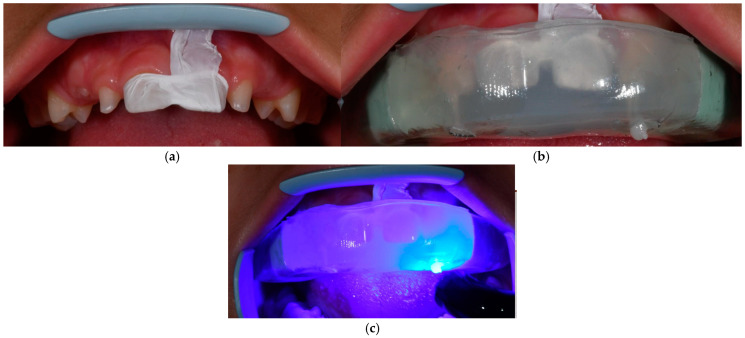
(**a**) The upper central incisors were isolated with Teflon to protect them from the flowable composite injection. (**b**) Silicon index in the patient’s mouth. It can be seen how the transparent silicon allows the clinician to see how the composite flows into the mould. (**c**) Light-curing procedure.

The final mould was fabricated once aesthetic and functional outcomes were satisfactory. A thermally moulded plastic tray covered the silicone stent, created based on the wax-up (Figure 9). Teflon was used to isolate the upper central incisors to protect them from the composite flow during the procedure. The upper lateral incisors were etched, and a bonding agent was applied. The flowable composite material was gradually injected to shape the teeth within the silicone index. After light curing, the excess composite was removed to prevent plaque accumulation and gingival irritation. A meticulous polishing regimen was performed to ensure the durability and aesthetics of the restoration. The materials and techniques employed mirrored those described in Case 1 (Figure 10).

Thermo-printed retainers were delivered during the same clinical session.

**Figure 11:**
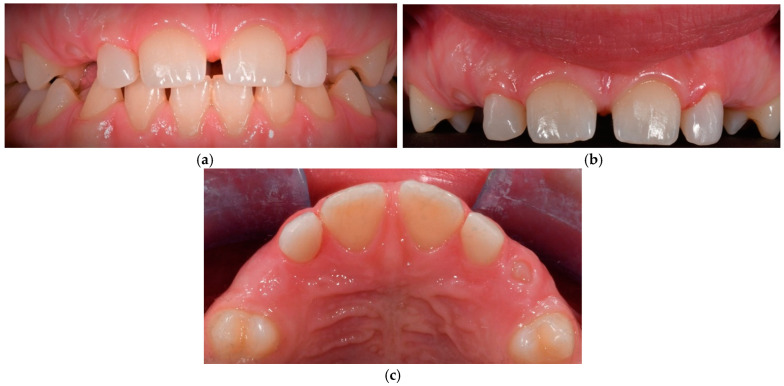
(**a**) Frontal view of final occlusion. (**b**) Detailed reshaping of teeth 12 and 22. (**c**) Occlusal view to highlight the palatal aspect of the reconstructions on 12 and 22.

A two-year follow-up revealed excellent outcomes, with no signs of bleeding, probing depth abnormalities, occlusal issues, or changes in tooth shape or colour. The patient tolerated the procedure well (Figure 11).

## 3. Discussion

The injectable technique is a straightforward treatment option for patients seeking aesthetic enhancement, particularly for minor re-anatomization needs [20]. These case reports highlight the practical application of the injectable composite resin technique for reshaping tooth form in young patients. This technique provides a cost-effective alternative for individuals who may prefer something other than expensive and invasive ceramic veneers. A significant advantage of composites over ceramics is their ease of repair or replacement. Moreover, composite restorations can be polished during subsequent interventions [21].

Recent advancements in flowable composites have significantly improved their mechanical properties, wear resistance, strength, polishability, and transparency [22,23]. High-load-capacity fluid resins exhibit enhanced flexural strength, resilience, modulus of elasticity [24,25], wear resistance, and polishability compared to conventional composites [26,27]. High filler content (69 wt%) contributes to uniform filler dispersion, resulting in wear-resistant restorations [28].

The successful application of the IMT requires meticulous tooth surface isolation without using rubber dam clamps to prevent interference with the thermo-formed tray. A thermo-printed tray (1 mm thickness) with support areas on non-restored teeth ensures precise moulding.

Heating the composite is critical to achieving optimal viscosity and consistency. A slight gingival overflow prevents air entrapment and ensures comprehensive material distribution in interproximal and marginal regions. Extended light-curing polymerisation is necessary to address physical obstructions and gaps between the light source and material [29]. Post-moulding rubber polishers safeguard the tooth shape. Restoration success depends on patient-specific risk factors, including age, parafunctional habits, and restoration size.

Photopolymerisation’s effect on restoration durability has been extensively studied [30,31]. Variations among resins from different manufacturers do not significantly impact restoration longevity, allowing clinicians to select materials based on preference [32,33]. This study used G-ænial Universal Injectable (GC Corporation), following manufacturer guidelines and supported by academic sources [7,34,35]. G-ænial Universal Injectable exhibited reduced Streptococcus mutans adherence due to its smoother surface. Sof-Lex Spirals enhanced surface smoothness [36].

Meticulous composite placement within the gingival sulcus prevents inflammation or gingivitis. A mechanical barrier prevents subgingival composite flow, mitigating biological complications. Maintaining supragingival margins on the wax-up eliminates the need for retraction cords and ensures effective treatment while minimising inflammation risk [10].

Careful case selection is paramount. Ideal candidates for the injectable technique include patients with minimal diastemas, small spaces, or dental retrusion [37]. The current cases exemplify irregular tooth size and morphology, demonstrating the suitability of the IMT for additive approaches.

## 4. Conclusions

- The IMT represents an optimal conservative treatment approach when an additive strategy can benefit the patient.
- Meticulous technique application, detailed pre-treatment planning, prudent case selection, and adherence to precise procedural and polishing protocols can achieve consistent and predictable outcomes.
- The IMT has demonstrated its utility in pediatric patients at the beginning and completion of orthodontic treatment.
- This technique is a valuable tool for orthodontists, facilitating the finalisation of treatment, addressing Bolton index discrepancies, and correcting tooth shape irregularities.
- Functional and aesthetic enhancements are achievable through the IMT, resulting in improved orthodontic outcomes and a more pleasing smile for patients.
- The integration of digital workflows enables clinicians to streamline clinical sessions by delivering thermo-printed retainers or aligners during the same appointment as the IMT, providing economic and organisational benefits.
- Further clinical studies are warranted to establish the long-term effectiveness of the technique.

## Data Availability

All data supporting this study’s findings are available from the corresponding author upon request.

## References

[1] Tin-Oo M.M., Saddki N., Hassan N. (2011). Factors influencing patient satisfaction with dental appearance and treatments they desire to improve aesthetics. BMC Oral Health.

[2] de Couto Nascimento V., de Castro Ferreira Conti A.C., de Almeida Cardoso M., Valarelli D.P., de Almeida-Pedrin R.R. (2016). Impact of orthodontic treatment on self-esteem and quality of life of adult patients requiring oral rehabilitation. Angle Orthod..

[3] Calamia J.R., Calamia C.S. (2007). Porcelain laminate veneers: Reasons for 25 years of success. Dent. Clin. N. Am..

[4] Turgut S., Bagis B. (2011). Colour stability of laminate veneers: An in vitro study. J. Dent..

[5] Beier U.S., Dumfahrt H. (2014). Longevity of silicate ceramic restorations. Quintessence Int..

[6] Demarco F.F., Collares K., Correa M.B., Cenci M.S., Moraes R.R.D., Opdam N.J. (2017). Should my composite restorations last forever? Why are they failing?. Braz. Oral Res..

[7] Terry D., Powers J. (2014). Using injectable resin composite: Part two. Int. Dent. Afr..

[8] Terry D.A. (2017). Restoring with flowables. Stomatol. EDU J..

[9] Terry D.A., Powers J.M., Blatz M.B. (2018). The inverse injection layering technique. J. Cosmet. Dent..

[10] Geštakovski D. (2019). The injectable composite resin technique: Minimally invasive reconstruction of esthetics and function. Quintessence Int..

[11] Gia N.R.Y., Sampaio C.S., Higashi C., Sakamoto A.S., Hirata R. (2021). The injectable resin composite restorative technique: A case report. J. Esthet. Restor. Dent..

[12] Pontons-Melo J.C., Atzeri G., Collares F.M., Hirata R. (2019). Cosmetic recontouring for achieving anterior esthetics. Int. J. Esthet. Dent..

[13] Pontons-Melo J.C., Pizzatto E., Furuse A.Y., Mondelli J. (2012). A conservative approach for restoring anterior guidance: A case report. J. Esthet. Restor. Dent..

[14] Coachman C., De Arbeloa L., Mahn G., Sulaiman T., Mahn E. (2020). An improved direct injection technique with flowable composites. a digital workflow case report. Oper. Dent..

[15] Valeri C., Quinzi V., Di Giandomenico D., Fani E., Leonardi R., Marzo G. (2023). Teledentistry: A bibliometric analysis of the scientific publication’s trend. Digit. Health.

[16] Coachman C., Calamita M. (2012). Digital smile design: A tool for treatment planning and communication in esthetic dentistry. Quintessence Dent. Technol..

[17] Canova F.F., Oliva G., Beretta M., Dalessandri D. (2021). Digital (r) evolution: Open-source software for orthodontics. Appl. Sci..

[18] Sampaio C.S., Puppin-Rontani J., Tonolli G., Atria P.J. (2021). Workflow of digitally guided direct composite resin restorations using open source software and 3d printing: A clinical technique. Quintessence Int..

[19] Riley D., Barber M., Kienle G., Aronson J., von Schoen-Angerer T., Tugwell P., Kiene H., Helfand M., Altman D., Sox H. (2017). Care 2013 explanations and elaborations: Reporting guidelines for case reports. J. Clin. Epidemiol..

[20] Terry D.A., Powers J.M. (2014). A predictable resin composite injection technique, part i. Dent. Today.

[21] Patankar R.C., More V., Jadhav R., Sabane A., Kadam P., Gachake A. (2022). Comparative evaluation of flexural strength of denture base resin materials processed using compression molding technique, injection molding technique, and computer-aided design cam technique: An in vitro study. Dent. Res. J..

[22] Boruziniat A., Gharaee S., Shirazi A.S., Majidinia S., Vatanpour M. (2016). Evaluation of the efficacy of flowable composite as lining material on microleakage of composite resin restorations: A systematic review and meta-analysis. Quintessence Int..

[23] Szesz A., Parreiras S., Martini E., Reis A., Loguercio A. (2017). Effect of flowable composites on the clinical performance of non-carious cervical lesions: A systematic review and meta-analysis. J. Dent..

[24] Prabhakar A., Madan M., Raju O. (2003). The marginal seal of a flowable composite, an injectable resin modified glass ionomer and a compomer in primary molars–an in vitro study. J. Indian. Soc. Pedod. Prev. Dent..

[25] Sumino N., Tsubota K., Takamizawa T., Shiratsuchi K., Miyazaki M., Latta M.A. (2013). Comparison of the wear and flexural characteristics of flowable resin composites for posterior lesions. Acta Odontol. Scand..

[26] Ujiie M., Tsujimoto A., Barkmeier W.W., Jurado C.A., Villalobos-Tinoco J., Takamizawa T., Latta M.A., Miyazaki M. (2020). Comparison of occlusal wear between bulk-fill and conventional flowable resin composites. Am. J. Dent..

[27] Imai A., Takamizawa T., Sugimura R., Tsujimoto A., Ishii R., Kawazu M., Saito T., Miyazaki M. (2019). Interrelation among the handling, mechanical, and wear properties of the newly developed flowable resin composites. J. Mech. Behav. Biomed. Mater..

[28] Maroulakos G., Maroulakos M.P., Tsoukala E., Angelopoulou M.V. (2021). Dental reshaping using the composite resin injection technique after dental trauma and orthodontic treatment. J. Dent. Child..

[29] Ammannato R., Ferraris F., Marchesi G. (2015). The “index technique” in worn dentition: A new and conservative approach. Int. J. Esthet. Dent..

[30] Balagopal S., Geethapriya N., Anisha S., Hemasathya B.A., Vandana J., Dhatshayani C. (2021). Comparative evaluation of the degree of conversion of four different composites polymerized using ultrafast photopolymerization technique: An in vitro study. J. Conserv. Dent. JCD.

[31] Demarco F.F., Cenci M.S., Montagner A.F., de Lima V.P., Correa M.B., Moraes R.R., Opdam N.J. (2022). Longevity of composite restorations is definitely not only about materials. Dent. Mater..

[32] Rodolpho P.A.D.R., Rodolfo B., Collares K., Correa M.B., Demarco F.F., Opdam N.J., Cenci M.S., Moraes R.R. (2022). Clinical performance of posterior resin composite restorations after up to 33 years. Dent. Mater..

[33] Moraes R.R., Cenci M.S., Moura J.R., Demarco F.F., Loomans B., Opdam N. (2022). Clinical performance of resin composite restorations. Curr. Oral Health Rep..

[34] Jang J., Park S., Hwang I. (2015). Polymerization shrinkage and depth of cure of bulk-fill resin composites and highly filled flowable resin. Oper. Dent..

[35] Kitasako Y., Sadr A., Burrow M., Tagami J. (2016). Thirty-six month clinical evaluation of a highly filled flowable composite for direct posterior restorations. Aust. Dent. J..

[36] Vulović S., Stašić J.N., Ilić J., Todorović M., Jevremović D., Milić-Lemić A. (2023). Effect of different finishing and polishing procedures on surface roughness and microbial adhesion on highly-filled composites for injectable mold technique. J. Esthet. Restor. Dent..

[37] Geštakovski D. (2021). The injectable composite resin technique: Biocopy of a natural tooth-advantages of digital planning. Int. J. Esthet. Dent..

